# Duodenal Leiomyoma Causing Gastrointestinal Bleeding: A Case Report

**DOI:** 10.7759/cureus.104114

**Published:** 2026-02-23

**Authors:** Haitam Yousfi, Mohamed Sinaa, Ayman Bijbij, Alae Chakir, Taoufik Lamsiah

**Affiliations:** 1 Gastroenterology, Moulay Ismail Military Hospital, Meknes, MAR; 2 Pathology, Moulay Ismail Military Hospital, Meknes, MAR; 3 Radiology, Moulay Ismail Military Hospital, Meknes, MAR

**Keywords:** duodenal, gastrointestinal bleeding, leiomyoma, neoplasm, rare

## Abstract

Duodenal leiomyomas are uncommon benign smooth muscle tumors that may remain clinically silent but can occasionally manifest with significant gastrointestinal bleeding, creating diagnostic difficulty because of their submucosal location. We report the case of a 72-year-old man who presented with acute gastrointestinal hemorrhage characterized by melena. Esophagogastroduodenoscopy revealed a bleeding submucosal lesion in the duodenum, and endoscopic biopsy was subsequently performed. Histopathological examination confirmed the diagnosis of duodenal leiomyoma. This case underscores the importance of considering duodenal leiomyoma in the differential diagnosis of acute gastrointestinal bleeding and highlights the diagnostic value of endoscopic biopsy in establishing a definitive histological diagnosis.

## Introduction

Small bowel primary neoplasms are uncommon; they represent less than 5% of all gastrointestinal tract neoplasms [1]. The most prevalent benign small bowel tumors are leiomyomas. It is uncommon for leiomyomas to occur in the duodenum, but they usually occur in the jejunum and then the ileum [2]. The majority of the lesions are small, asymptomatic, and are frequently discovered incidentally during endoscopy [3]. There are not many case reports of symptomatic duodenal leiomyomas in the literature. Herein, we report the case of a 72-year-old man who presented with acute gastrointestinal bleeding caused by a duodenal leiomyoma. This report highlights an unusual clinical presentation of these rare benign tumors and emphasizes the importance of considering them in the differential diagnosis of upper gastrointestinal hemorrhage.

## Case presentation

**Figure 1:**
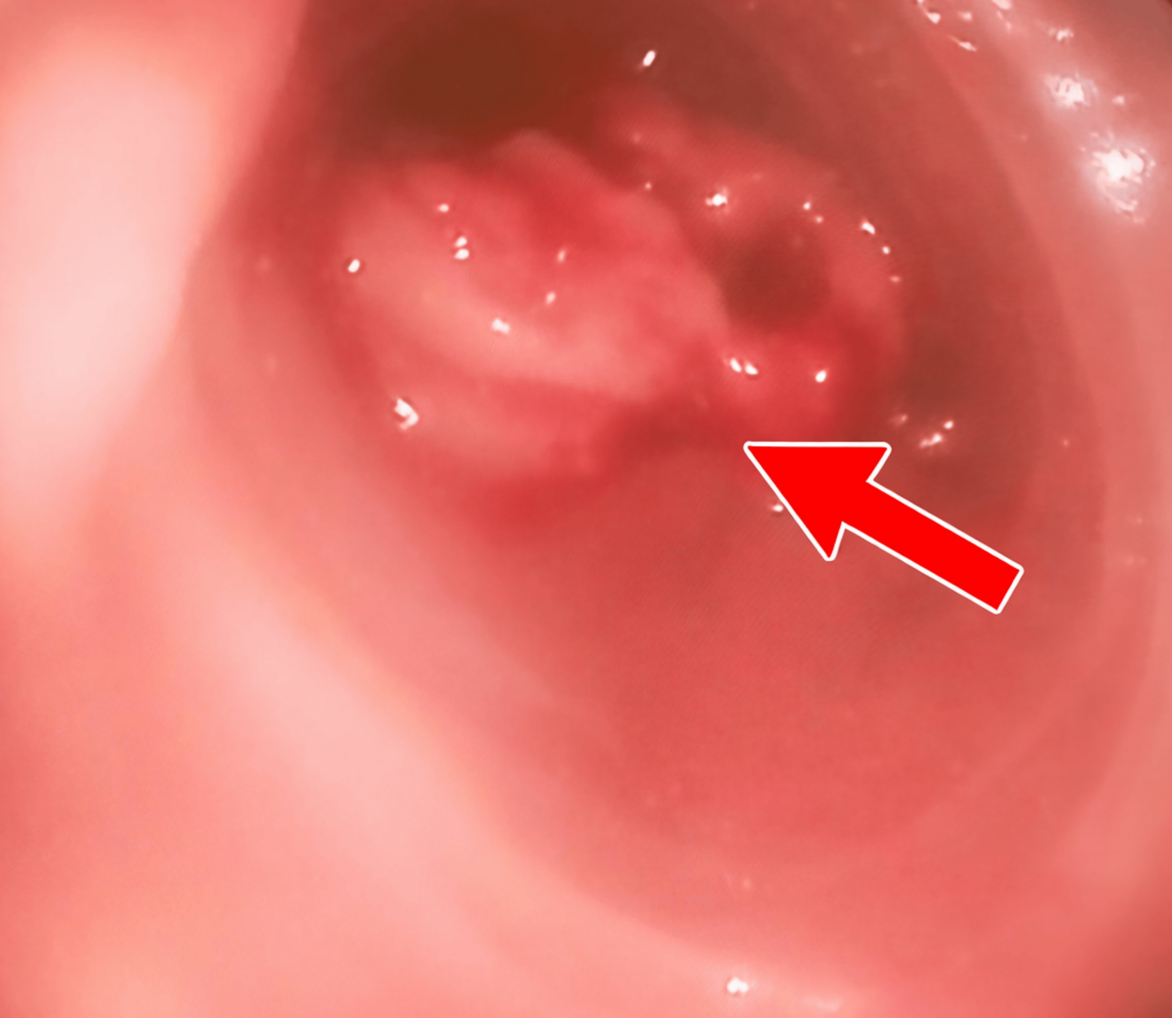
Endoscopic image showing a bleeding, ulcerated, and exophytic lesion located in the second portion of the duodenum

A 72-year-old man was admitted to the emergency department for dark stools evolving over five days, with a six-month history of epigastric pain. On admission, the patient was tachycardic (heart rate 106 beats per minute), hemodynamically stable, and exhibiting mild cutaneous and mucous membrane pallor. Clinical examination revealed mild epigastric tenderness, and digital rectal examination confirmed black stools. The remainder of the physical examination was unremarkable. Biological investigations revealed a hemoglobin level of 8.5 g/dL, a mean corpuscular volume (MCV) of 72 fL, with a normal leukocyte count. Electrolyte balance and renal function were within normal limits. An upper gastrointestinal endoscopy was performed, revealing a bleeding ulcerated and exophytic lesion located in the second portion of the duodenum (Figure 1).

**Figure 2:**
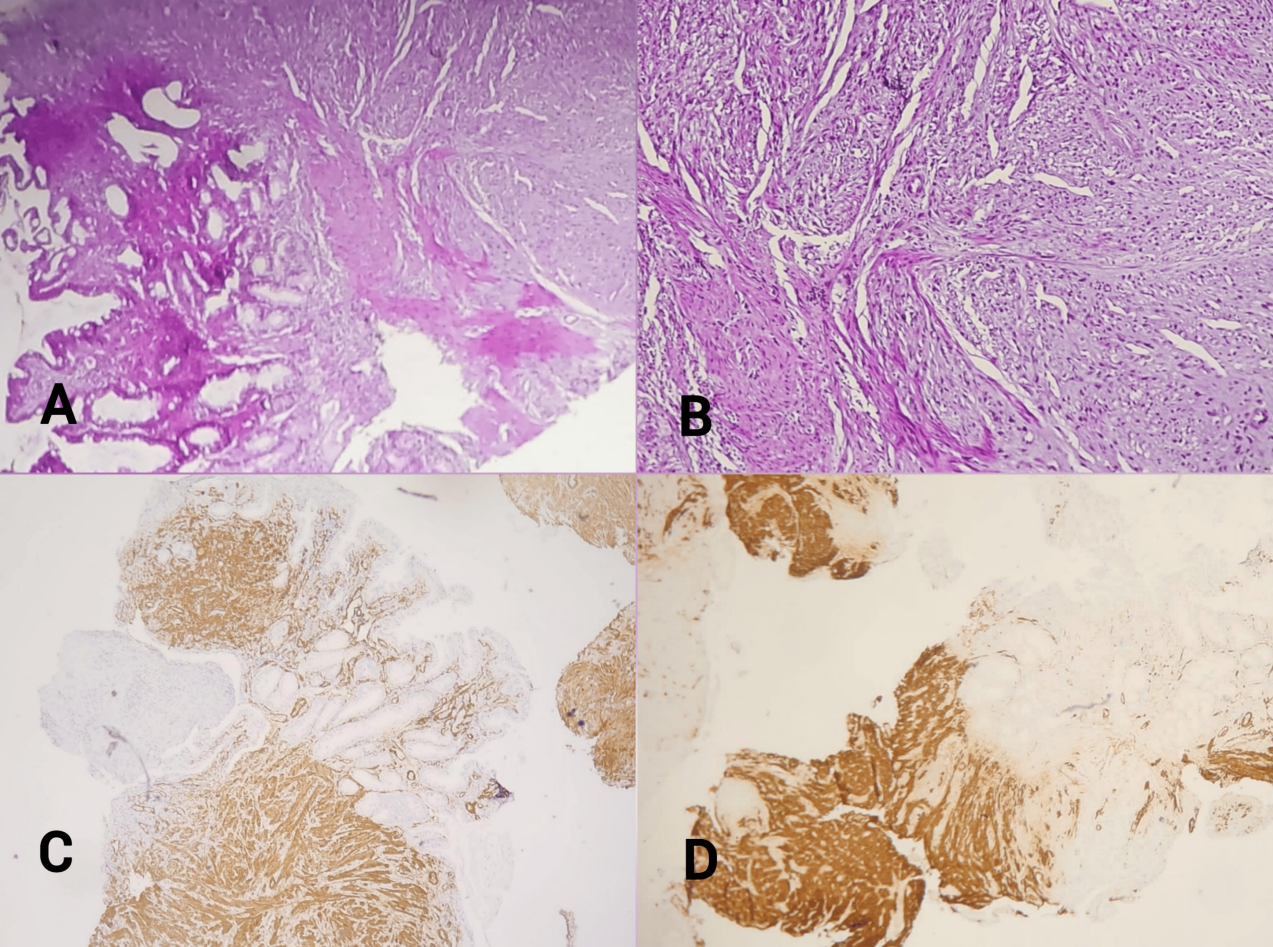
Histopathological examination and immunohistochemical analysis A, B: Microscopic images showing a duodenal mucosa with a benign tumor proliferation consisting of smooth muscle bundles in the chorion. No cytonuclear atypia or mitoses seen (hematoxylin & eosin stain, magnification ×200). C: Immunohistochemical microscopic image showing intense tumor staining by anti-smooth muscle actin antibodies (magnification ×100). D: Immunohistochemical microscopic image showing intense tumor staining by anti-H-caldesmon antibodies (magnification ×200).

Endoscopic biopsies were obtained. Histopathological examination and immunohistochemical analysis were consistent with a duodenal leiomyoma, showing tumor cell positivity for smooth muscle actin (SMA) and H-caldesmon, and negativity for CD117, CD34, S100 protein, and desmin (Figure 2).

**Figure 3:**
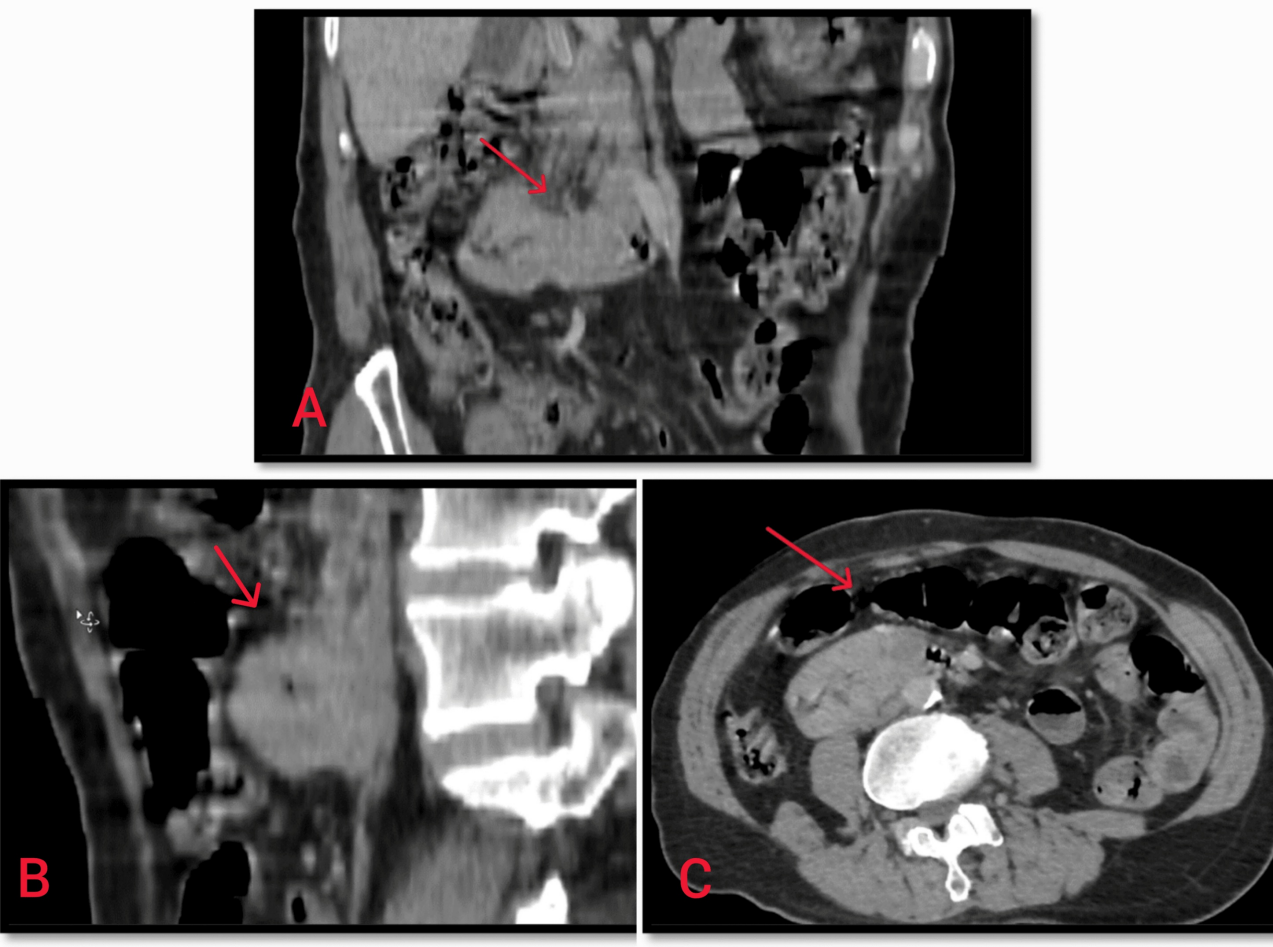
Contrast-enhanced abdominal CT with multiplanar reconstructions. A regular, circumferential, symmetric prestensotic endoluminal duodenal wall thickening is centered at the D2–D3 junction (red arrow), without suspicious locoregional lymphadenopathy and without CT signs of upstream obstruction. (A) Coronal multiplanar reconstruction (MPR): circumferential wall thickening at the D2-D3 junction (red arrow). (B) Sagittal MPR: segmental prestensotic endoluminal thickening involving the D2-D3 junction (red arrow). (C) Axial CT: confirmation of symmetric circumferential thickening at the D2-D3 junction (red arrow).

An abdominal contrast-enhanced computed tomography scan demonstrated a regular, symmetrical, prestenotic endoluminal thickening involving the D2-D3 junction, with no suspicious locoregional lymphadenopathy and no upstream digestive obstructive syndrome (Figure 3).

After a multidisciplinary discussion with the surgical team, surgical management was indicated; however, the patient declined any surgical intervention.

## Discussion

Leiomyomas are benign smooth muscle neoplasms that typically originate in tissues rich in smooth muscle fibers. They most commonly occur in the uterus [4,5]. In the upper gastrointestinal tract, leiomyomas are frequently located in the stomach (61; 5%) and jejunum (19; 5%) but rarely in the duodenum (5%) [6]. Duodenal leiomyomas typically present in patients in their sixth to seventh decades of life and show a slight male predominance [7]. In our case, the patient was a 72-year-old male. Duodenal leiomyomas are generally asymptomatic, although larger tumors may manifest with gastrointestinal bleeding, abdominal pain, an abdominal mass, or obstructive symptoms [3]. The patient presented in our article had suffered from epigastric pain for six months and a recent history of melena.

Endoscopy represents the most sensitive modality for the detection of these rare tumors. However, endoscopic biopsy may yield normal duodenal mucosa, as the lesion is typically located within the submucosa. Endoscopic ultrasonography provides greater sensitivity for the visualization and characterization of submucosal tumors [7,8,9].

Computed tomography (CT) plays a crucial role in identifying the duodenal wall origin of the lesion and evaluating its extent. On CT imaging, leiomyomas typically appear as well-circumscribed masses with homogeneous density and smooth borders [10,11].

The differential diagnosis of a duodenal submucosal mass includes several benign and malignant entities. The most important consideration is the gastrointestinal stromal tumor, which represents the most common mesenchymal tumor of the gastrointestinal tract and may present with bleeding. Other differential diagnoses include leiomyosarcoma, a malignant smooth muscle neoplasm characterized by marked cytologic atypia and high mitotic activity; neuroendocrine tumor, which may occur in the duodenum and present with bleeding or obstruction; Brunner gland hyperplasia; and duodenal adenocarcinoma.

Histopathological and immunohistochemical evaluation is essential to establish the definitive diagnosis and to differentiate leiomyoma from these entities [12]. Histopathological examination reveals mature smooth muscle cells associated with stromal hyalinization, areas of coagulative necrosis, and a low mitotic index. Immunohistochemical analysis demonstrates positivity for smooth muscle actin (SMA) and desmin or H-caldesmon, with negative staining for S-100, Ki-67, CD34, and HMB-45. Despite these features, the malignant potential of leiomyomas cannot be entirely excluded [5].

Surgery is considered the gold standard in the treatment of duodenal leiomyomas. The surgical strategy is determined by the size and location of the tumor, as well as the likelihood of malignancy suggested by imaging studies. Surgical options include local excision, segmental duodenal resection, and pancreaticoduodenectomy [6]. The prognosis of this tumor is favorable. In the case of radical surgery, local recurrence is rare.

## Conclusions

Duodenal leiomyomas are a rare but important cause of upper gastrointestinal bleeding. Although small bowel leiomyomas are known to bleed, duodenal involvement is uncommon and may clinically mimic more frequent conditions such as peptic ulcer disease or malignant submucosal tumors. Definitive diagnosis relies on histopathological and immunohistochemical evaluation to distinguish leiomyomas from entities such as gastrointestinal stromal tumors, which carry different prognostic implications. Complete surgical excision is curative, with an excellent long-term outcome.
